# Correction: Veeramani et al. Quantifying PD1 Saturation by PDL1 in Tumor Tissue Using a Novel RNA Aptamer-Based Assay. *Int. J. Mol. Sci.* 2026, *27*, 5469

**DOI:** 10.3390/ijms27167082

**Published:** 2026-08-07

**Authors:** Suresh Veeramani, Chaobo Yin, Nanmeng Yu, Kristen Coleman, Brian J. Smith, George J. Weiner

**Affiliations:** 1 Department of Internal Medicine, University of Iowa, Iowa City, IA 52242, USA; suresh-veeramani@uiowa.edu (S.V.); nanmeng-yu@uiowa.edu (N.Y.); 2 Holden Comprehensive Cancer Center, University of Iowa, Iowa City, IA 52242, USA; chaobo-yin@uiowa.edu (C.Y.); kristen-coleman@uiowa.edu (K.C.); brian-j-smith@uiowa.edu (B.J.S.); 3 Department of Biostatistics, University of Iowa, Iowa City, IA 52242, USA

There was an error in the original publication [1] in the Institutional Review Board Statement and the Conflicts of Interest section in the back matter of the manuscript. The corrected text appears below:

Institutional Review Board Statement: De-identified FFPE biospecimens used in this study were obtained from the Tissue Procurement Core (TPC) at the University of Iowa, which operates under IRB protocol #201708847 (Ethics Committee Name: Institutional Review Board, Human Subjects Office, University of Iowa; original approval date: 11 February 2017; renewed: 4 December 2026).

Conflicts of Interest: Author G.J.W. is the founder and CEO of LIRECAP, Inc.; S.V. has ownership interests in LIRECAP, Inc.

The authors state that the scientific conclusions are unaffected, as this information was in the original manuscript (in a different position). This correction was approved by the Academic Editor. The original publication has also been updated.
