# Supplementary material for: Quantifying PD1 Saturation by PDL1 in Tumor Tissue Using a Novel RNA Aptamer-Based Assay
Source: Int J Mol Sci. 2026 Jun 17;27(12):5469. doi: 10.3390/ijms27125469 (PMC13299433; doi:10.3390/ijms27125469)
Supplement: Supplementary file 1 [file ijms-27-05469-s001.zip › ijms-4315462-supplementary.pdf]

Supplementary Figures:

Figure S1: Number of unique aptamer sequences during SELEX

Data displaying a reduction in the number of unique RNA aptamer sequences with each round of SELEX, indicating progressive enrichment as rounds progressed.

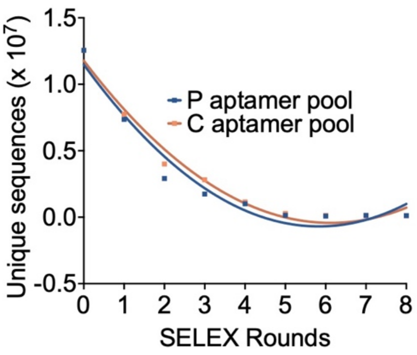

Figure S2: Cross-reactivity of aptamers with PDL2 and PD1-PDL2 complex

(A) P17 and (B) C18 aptamers display a preferential binding pattern towards PD1 and PD1-PDL2 complex, respectively, like the binding pattern with PD1-PDL1 complex, suggesting a structural similarity between the complexes suggesting the C aptamer binds to both the PD1-PDL1 Complex and the PD1-PDL2 Complex.

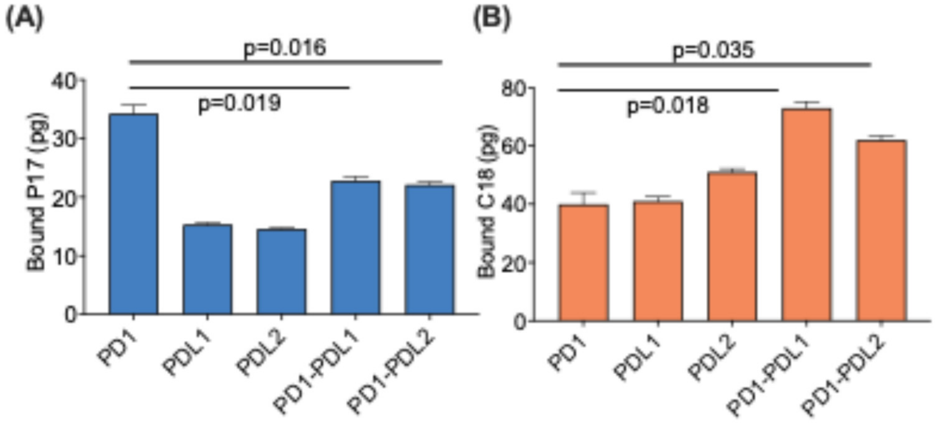

Figure S3: Specificity of aptamers and probes for PD1 LIRECAP assay

(A) P17 (blue) and C18 (orange) aptamers do not compete for binding to PD1 or PD1-PDL1 Complex and do not cross-block each other when added together in the PD1 LIRECAP assay. (B) TaqMan probe designed for P17 show high specificity to P17 aptamer, not C18 aptamer. (C) TaqMan probe designed for C18 show high specificity to C18 aptamer, not P17 aptamer.

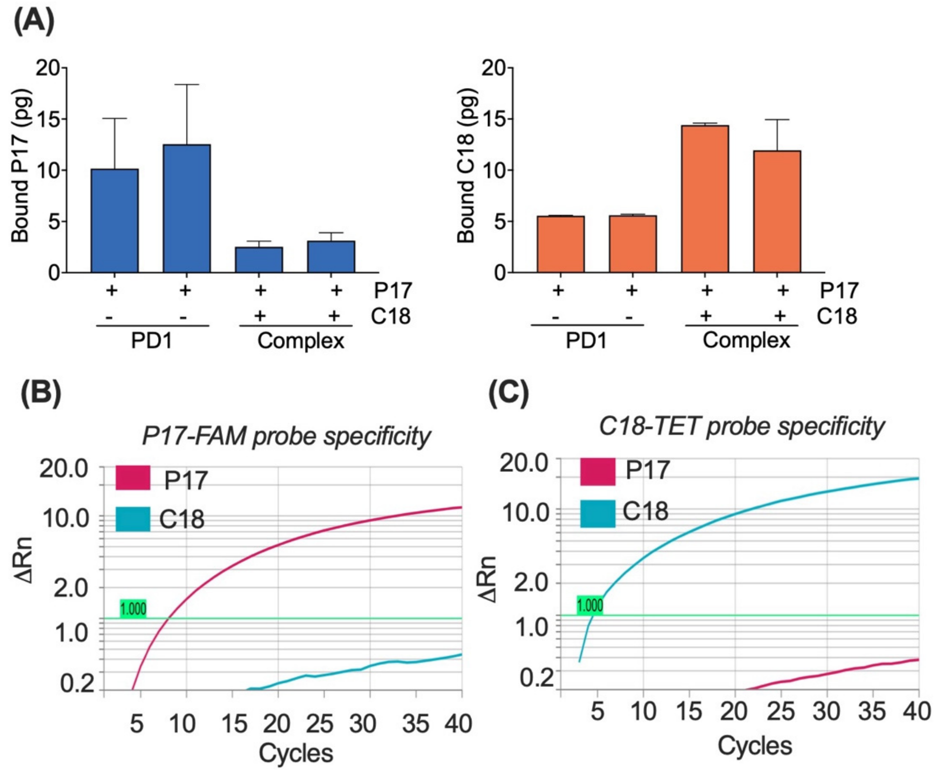

Supplementary tables:

Table S1: Starting RNA library and SEL2 PCR primer sequences

Name	*Sequence (5' --> 3')*
Starting RNA library*	5' – GGGAGGACGAUGCGG – $(N)_{20}$ – CAGACGACUCGCCCGA – 3'
SEL2 5' primer	5'-TAATACGACTCACTATAGGGAGGACGATGCGG-3'
SEL2 3' primer**	5'-mUmCGGGCGAGTCGTCTG-3'

* RNA was transcribed from the synthetic DNA strand using rRfY NTP mix.

**The reverse primer was modified with 2'O-Me in the first two nucleotides (5') to improve the transcription accuracy[36]

Table S2: SELEX binding conditions

<table>
<tr><th>SELEX rounds</th><th>Preclearing*</th><th>Post-clearing**</th><th>[RNA]</th><th>[Target protein]***</th><th>Binding time</th></tr>
<tr><td>1 & 2</td><td>200 nM His-HSA + human IgG1</td><td>N/A</td><td>2 μm</td><td>200 nM</td><td>120 min</td></tr>
<tr><td>3 & 4</td><td>200 nM His-HSA + human IgG1</td><td>N/A</td><td>2 μm</td><td>200 nM</td><td>60 min</td></tr>
<tr><td>5 & 6</td><td>200 nM His-HSA + human IgG1</td><td rowspan="4">1. 100 nM PDL1 for post-clearing PD1 SELEX
2. 100 nM PD1 followed by PDL1 for post-clearing Complex SELEX</td><td>1 μm</td><td>200 nM</td><td>45 min</td></tr>
<tr><td>7</td><td>300 nM His-HSA + His-UbQ + human IgG1</td><td>500 nM</td><td>50 nM</td><td>45 min</td></tr>
<tr><td>8</td><td>300 nM His-HSA + His-UbQ + human IgG1</td><td>500 nM</td><td>50 nM</td><td>30 min</td></tr>
<tr><td>9</td><td>500 nM His-HSA + His-UbQ + human IgG1</td><td>100 nM</td><td>25 nM</td><td>15 min</td></tr>
</table>

* Preclearing of RNA pool was performed before binding with target proteins to remove aptamers non-specifically binding to tags (6X His or Fc) and proteins

** Post-clearing of RNA pool was performed after the binding step with target proteins to remove aptamers that cross-reacted between PD1 and PDL1

*** Target proteins concentrations provided are based on the PD1 protein used for PD1 SELEX. For Complex SELEX, PDL1 was added to PD1-coated beads at enough concentration to create at least 75% PD1 saturation.[24]

Table S3: Full RNA sequence of the of the selected aptamers

Aptamer name	*Variable region (5' --> 3')*
P10	ATAGTGAGTCGTGGCCGCCT
P17	ATAGTGAGTCGTGGCCGCCCT
P150	TCCTGTCGTCTGTTCGTCCT
C18	CTCTGTCGTCTGTTCGTCCC
C52	ATAGTCGTTCCCTTCGTCCC
C113	ACCGTGGAGTATTGCCGCCC

Table S4: Fold enrichment of selected candidates from PD1 SELEX and Complex SELEX over successive SELEX rounds

Aptamer name	*Fold enrichment in respective SELEX (normalized to Rd 0)*				
	Rd 1	*Rd 3*	*Rd 5*	*Rd 7*	*Rd 8*
P10	0.4	31.8	42.2	283.5	1413.3
P17	0.3	6.8	22.6	152.7	661.5
P150	1.0	174.5	73.3	218.7	194.3
C18	0.4	1.3	134.9	541.8	1244.1
C52	0.5	7.2	99.2	697.4	768.4
C113	1.0	0	98.3	320.9	240.5

Table S5: Primer and Probe sequences used for PD1 LIRECAP assay

Name	*Sequence (5' --> 3')*
Forward qPCR primer	TATAGGGAGGACGATGCGG
Reverse qPCR primer	TCGGGCGAGTCGTCT
TaqMan probe for P17 aptamer	FAM-AGGGCGGCCACGACTCACTAT
TaqMan probe for C18 aptamer	TET-CTCTGTCGTCTGTTCGTCCCCA

Table S6: Co-culturing Jurkat-PD1 and Raji-Null cells to obtain varying ranges of PD1 saturation by PDL1

PDL1 to PD1 (%)	***Jurkat-PD1***	***Raji-Null***	***Raji-PDL1***
0	*100*	*100*	*0*
6.25	*100*	*93.75*	*6.25*

12.5	*100*	*87.5*	*12.5*
25	*100*	*75*	*25*
50	*100*	*50*	*50*
100	*100*	*0*	*100*

Table S7: Immunological characteristics of Human FFPE sarcoma biospecimens

	**Sample Name**	**IHC data**		
		PDL1	Immune cells	CD3
T cell^{high}PDL1^{low}	A	-	+	+
	B	-	+	+
	C	-	+	+
T cell^{high}PDL1^{high}	D	+	+	+
	E	+	+	+
	F	+	+	+
